# Correction: Experimental and theoretical study of magnetohydrodynamic ship models

**DOI:** 10.1371/journal.pone.0186166

**Published:** 2017-10-04

**Authors:** David Cébron, Sylvain Viroulet, Jérémie Vidal, Jean-Paul Masson, Philippe Viroulet

Labels are missing from Fig 2 and Fig 7. Please see the corrected Figs 2 and 7 here.

**Fig 2:**
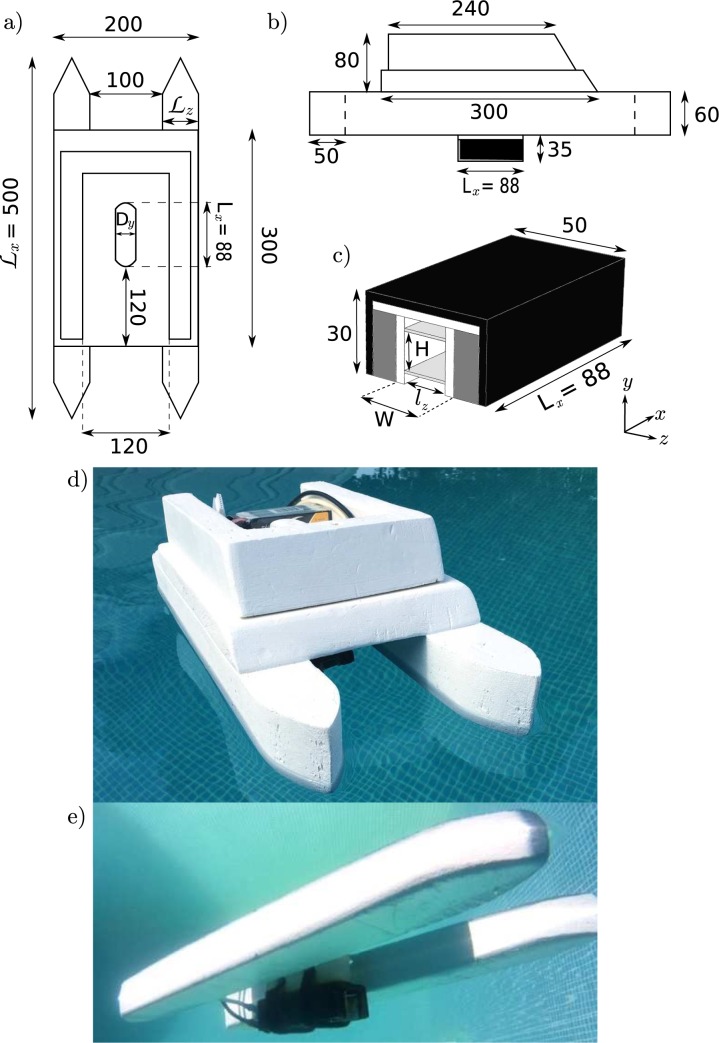
**Schematic representation of top (a) and side (b) view of the ship (length are in millimeters).** (c) Diagram of the thruster used to propel the ship. The black part represents the magnetic bridge, dark gray part the magnets, light gray part the electrodes and the white part the plastic used to isolate the electric circuit. Pictures (d) and (e) are aerial and underwater view of the ship respectively. The 6s LiPo Battery is visible on picture (d). Note that for aesthetics reasons, pictures have been taken in a swimming pool and not in the actual experimental tank.

**Fig 7:**
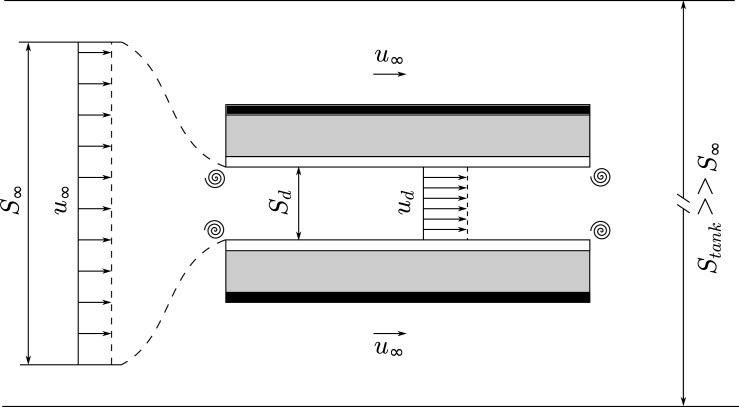
Sketch of the thruster and the different velocities taken into account in this study. Four vortices are displayed to represent the singular head loss at the entrance and exit of the thruster.
