# Supplementary material for: Experimental and theoretical study of magnetohydrodynamic ship models
Source: PLoS One. 2017 Jun 30;12(6):e0178599. doi: 10.1371/journal.pone.0178599 (PMC5493298; doi:10.1371/journal.pone.0178599)
Supplement: S1 Appendix — (ZIP) [file pone.0178599.s001.zip › S1_Appendix.pdf]

## Discussion of electrolysis complications

In many electrolysis reactions, a limiting current density, i.e. a maximum value for $j$ in $\delta U = A_0 \ln \frac{j}{j_0}$, is reached, indicating that the electrolysis have consummated all the reactant present in the thin diffusion layer in contact with the electrodes. Here, this limiting diffusion regime can only appear for $Na^+$ and $Cl^-$ because the other reactants are the solvent (water) and the electrodes [1]. Moreover, for $C = 35\,\mathrm{kg.m^{-3}}$, this limiting diffusion regime is reached for $j \sim 10^3\,\mathrm{A.m^{-2}}$ in a cell, whereas the limiting current density of a turbulent flowing electrolyte is rather $j \sim 10^7\,\mathrm{A.m^{-2}}$ , which is far above the typical current density $j \sim 10^3 - 10^4\,\mathrm{A.m^{-2}}$ considered in this work [2, 3]. This maximum value for $j$ is thus not relevant for our MHD thruster.

Since the MHD thruster imposes a magnetic field in the fluid, one can wonder if the presence of a magnetic field modifies the picture. The Lorentz force acting on the charge carrier is actually modified by the presence of a magnetic field, giving an effective reduction in mobility for motion perpendicular to the magnetic field. The electrical conductivity in this direction (Pedersen conductivity) is thus reduced. This so-called magnetoresistance can be estimated by using a usual Drude model of electrical conduction, which shows that the resistance if increased by a factor $1 + (\mu^* B)^2$, where $\mu^*$ is the charge carrier mobility and $B$ the magnetic field (see [4] for details). In our experiments, $\mu^* B$ is typically of order $10^{-7}$ at most, and this effect is thus largely negligible.

Since the MHD thruster pumps water, one can also wonder how a non-zero fluid velocity in the electrolysis cell (i.e. a moving ship) modifies the picture, and how this affects the performances of the MHD thruster. First, the Tafel slope $A_0$ is increased by a non-zero fluid velocity, probably because of hydrodynamic boundary layer resistance enhancement [5]. Second, following [6], one can notice that the presence of bubbles downstream is dependent on both current density and fluid velocity. Indeed, hydrogen bubbles exist downstream only below a certain critical fluid velocity $u_c$, which increases with the imposed current density (e.g. $u_c = 3.5\,\mathrm{m.s^{-1}}$ for $j = 250\,\mathrm{A.m^{-2}}$, $u_c = 7\,\mathrm{m.s^{-1}}$ for $j = 500\,\mathrm{A.m^{-2}}$). It seems thus that the hydrogen goes into solution rapidly for large enough fluid velocities [6]. Second, a large enough fluid velocity also allows to sweep the electrolysis bubbles downstream, avoiding to accumulate electrolysis bubbles on the electrodes. These bubbles can indeed form large insulating gas pockets, which would drastically reduce the electric current. The bubbles are $0.1 - 0.5\,\mathrm{mm}$ in diameter, which gives a rise velocity in water of $1 - 5\,\mathrm{cm.s^{-1}}$, and most of the bubbles are thus swept out of the channel before rising the top electrode as soon as the fluid velocity is larger than $\sim 0.2\,\mathrm{m.s^{-1}}$ (see [7] for details). Note also that [2] show that the electrolysis bubbles do not affect the flow. Since the fluid average velocity $u_d$ is systematically larger than $0.2\,\mathrm{m.s^{-1}}$ in our measurements, electrolysis bubbles do not need to be taken into account.

## References

1. Mathon P, Nouri A, Alemany A, Chopart J, Sobolik V, Baaziz D. Electro-chemical processes controlled by high magnetic fields: application to MHD sea water propulsion. Magnetohydrodynamics c/c of Magnitnaia Gidrodinamika. 2009;45(2):281–288.

2. Boissonneau P, Thibault J. Experimental analysis of couplings between electrolysis and hydrodynamics in the context of MHD in seawater. Journal of Physics D: Applied Physics. 1999;32(18):2387.

3. Boissonneau P, Thibault JP. Sea water MHD: electrolysis and gas production in flow. In: Transfer Phenomena in Magnetohydrodynamic and Electroconducting Flows. Springer; 1999. p. 251–266.

4. Tronel-Peyroz E, Olivier A. Application of the Boltzman equation to the study of electrolytic solution in the presence of electric and magnetic fields. Physico-Chemical Hydrodynamics. 1982;3:251–265.

5. Petrick M, Libera J, Bouillard J, Pierson E, Hill D. Results from a large-scale MHD propulsion experiment. Argonne National Lab.; 1992.

6. Picologlou B, Doss E, Black D, Sikes WC. Experimental determination of magnetohydrodynamic seawater thruster performance in a two Tesla test facility. SAE Technical Paper; 1992.

7. Tempelmeyer KE. Electrical Characteristics of a Seawater MHD Thruster. DTIC Document; 1990.
