# Supplementary material for: Experimental and theoretical study of magnetohydrodynamic ship models
Source: PLoS One. 2017 Jun 30;12(6):e0178599. doi: 10.1371/journal.pone.0178599 (PMC5493298; doi:10.1371/journal.pone.0178599)
Supplement: S2 Appendix — (ZIP) [file pone.0178599.s002.zip › S2_Appendix.pdf]

α and β for the Hartmann flow

The coefficients (α, β) for an arbitrary flow profile u through a section S are defined by $\alpha = 1/S \cdot \int_S [u/\bar{u}]^3 \mathrm{d}\tau$ and $\beta = 1/S \cdot \int_S [u/\bar{u}]^2 \mathrm{d}\tau$, with $\bar{u}$ the mean flow velocity). For instance, a Poiseuille flow in a cylinder gives $(\alpha = 2, \beta = 4/3)$, and for a plug flow, $\alpha \approx \beta \approx 1$.

To investigate the effect of a magnetic field on (α, β) in a simple manner, a plane Poiseuille flow is considered in presence of a magnetic field. This so-called Hartmann flow [1] is thus the flow between two parallel plates separated by a distance W, with a uniform magnetic field B perpendicular to the planes. The velocity is then [1]

$$\frac{u}{\bar{u}} = H_a \frac{\cosh(H_a) - \cosh(H_a Z)}{H_a \cosh(H_a) - \sinh(H_a)}, \tag{1}$$

where $Z = z/(W/2)$, using an axis Oz, perpendicular to the planes, with an origin located at a distance $W/2$ from the planes. The Hartmann number H_a is given by $H_a = WB(\sigma/\eta)^{1/2}/2$, where σ is the fluid electrical conductivity, and η the fluid dynamic viscosity. Using the velocity (1) in $\beta = 1/2 \cdot \int_{-1}^{1} [u/\bar{u}]^2 \; dZ$ gives

$$\beta = \frac{H_a}{4} \frac{\sinh(2H_a) - 8\cosh(H_a)\sinh(H_a) + Q}{H_a^2 \cosh^2 H_a - H_a \sinh(2H_a) + \sinh^2 H_a} \tag{2}$$

with $Q = 2H_a[1 + 2\cosh^2 H_a]$. Similarly, $\alpha = NH_a^2/(12D)$ is obtained from $\alpha = 1/2 \cdot \int_{-1}^{1} [u/\bar{u}]^3 \; dZ$, with $N = 3H_a \cosh(3H_a) - \sinh(3H_a) + \cosh(H_a)[27H_a + 9\sinh(2H_a)] - \sinh(H_a)[27 + 18\cosh(2H_a)]$ and $D = H_a \cosh(H_a)[H_a^2 \cosh^2 H_a + 3\sinh^2 H_a] - \sinh(H_a)[\sinh^2 H_a + 3H_a^2 \cosh^2 H_a]$.

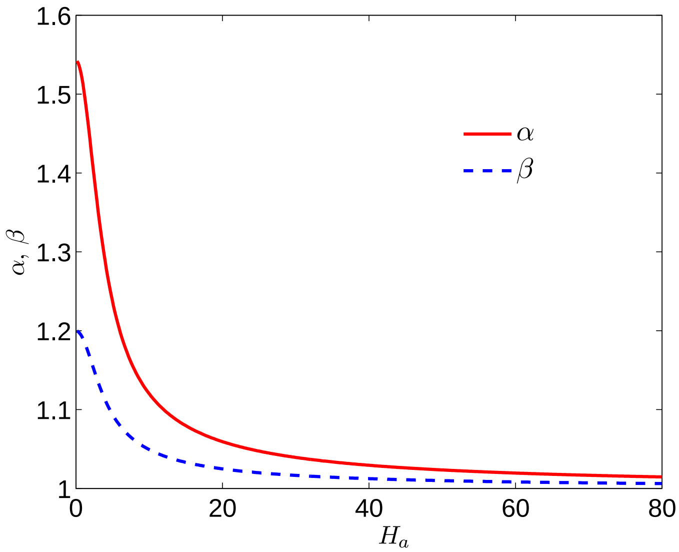

Fig 1. Evolution of the coefficients α and β with H_a.

The evolution of both α and β as a function of H_a is represented in Fig 1. Typical values of $\alpha = 54/35$ and $\beta = 6/5$ for the plane Poiseuille flow are recovered for $H_a = 0$.

Both coefficients tend towards 1 in the limit $H_a \gg 1$, where the flow tends to a uniform flow profile.

In our case, a typical value is $H_a = 2.5$, obtained for $W = 10\,\mathrm{cm}$, $B = 0.5\,\mathrm{T}$, $\sigma = 10\,\mathrm{S.m^{-1}}$, and $\eta = 10^{-3}\,\mathrm{Pa.s}$.

References

1. Hartmann J, Lazarus F. Hg dynamics. Levin & Munksgaard; 1937.
