# Supplementary material for: Experimental and theoretical study of magnetohydrodynamic ship models
Source: PLoS One. 2017 Jun 30;12(6):e0178599. doi: 10.1371/journal.pone.0178599 (PMC5493298; doi:10.1371/journal.pone.0178599)
Supplement: S3 Appendix — (ZIP) [file pone.0178599.s003.zip › S3_Appendix.pdf]

Analytical solutions for the thruster

In order to obtain expressions for the thruster optimal parameters, we aim at solving analytically

$$\begin{aligned} \beta_d &= \frac{S_w C_d(\lambda u_d)}{2 S_d}\lambda^2 + \beta_\infty \lambda, && (1)\\ U_0 &= E_0 + A_0 \ln I + RI + k u_d BH && (2)\\ IBH &= \mathcal{K}\left[1 + \mathcal{G}(u_d)\right] u_d^2. && (3)\end{aligned}$$

To do so, we rely on the three assumptions described in section 4.3. Assuming a constant C_d allows to uncouple Eq 1, leading to the solution

$$\lambda = \frac{u_\infty}{u_d} = \frac{S_d}{S_\infty} = \frac{S_d}{S_w C_d}\left[\sqrt{\beta_\infty^2 + \frac{2\beta_d S_w C_d}{S_d}} - \beta_\infty\right], \quad (4)$$

Thus, u_∞ is known as soon as u_d is obtained. The MHD thruster is thus fully governed by equations (2)-(3), and the two other assumptions allow to write them as

$$\begin{aligned} U_0 &= E_0 + RI + k u_d BH && (5)\\ IBH &= \mathcal{K}\left(1 + \tilde{\nu}/u_d\right) u_d^2, && (6)\end{aligned}$$

where $\tilde{\nu} = 64\,\nu\, L_x/[D_h^2\,(\alpha_d - \lambda^2\alpha_\infty + \xi(1-\lambda^2))]$. Solving equations (5)-(6) leads to

$$\begin{aligned} \frac{I}{I_{B\to 0}} &= 1 - \left(2\kappa - \frac{\tilde{\nu}}{u_{d,B\to 0}}\right)\left(\sqrt{1+\kappa^2} - \kappa\right) && (7)\\ \frac{u_d}{u_{d,B\to 0}} &= \sqrt{1+\kappa^2} - \kappa, && (8)\end{aligned}$$

with

$$\kappa = \frac{1}{2}\left(\frac{B}{B_{typ}}\right)^{3/2} + \frac{\tilde{\nu}}{2\,u_{d,B\to 0}}, \quad (9)$$

where $B_{typ} = [(U_0 - E_0)\mathcal{K}R/k^2]^{1/3}/H$, and

$$\begin{aligned} I_{B\to 0} &= (U_0 - E_0)/R && (10)\\ u_{d,B\to 0} &= \sqrt{(U_0 - E_0)BH/(\mathcal{K}R)}. && (11)\end{aligned}$$

Equations (10)-(11) are solutions of equations (5)-(6) when $\kappa = 0$, i.e. when the regular head loss and induced electric field are negligible ($\tilde{\nu} = 0$, $k = 0$). In this limit, the equations are uncoupled: I is fixed by Eq (5) and u_d by Eq (6). In the other limit ($RI \ll k u_d BH$, $\tilde{\nu} = 0$), the equations are also uncoupled and the thruster behaves as a current generator. Then u_d is given by Eq (5), and I by Eq (6), i.e.

$$\begin{aligned} u_{B\to\infty} &= (U_0 - E_0)/(kBH) && (12)\\ I_{d,B\to\infty} &= \mathcal{K}(U_0 - E_0)^2/(k^2(BH)^3), && (13)\end{aligned}$$

which allows to give a physical interpretation to κ with

$$2\,\kappa = \frac{u_{d,B\to 0}}{u_{B\to\infty}} = \sqrt{\frac{I_{B\to 0}}{I_{d,B\to\infty}}}. \quad (14)$$

When B is increased, $u_{d,B\to 0}$ increases via the term IBH, and $u_{B\to\infty}$ decreases because of the term $ku_d BH$. This shows that a magnetic field value B_{opt} should maximise u_d. Solving $\partial_B u_d = 0$ gives B_{opt} as

$$B_{opt} = \left[\frac{\mathcal{K}R(U_0 - E_0)}{2k^2 H^3}\right]^{1/3} = 2^{-1/3}\, B_{typ}, \tag{15}$$

where we have assumed $\tilde{\nu} = 0$ (negligible regular head loss). Under this assumption, and for the field (15)

$$u_d(B_{opt}) = \max_B u_d = \left[\frac{(U_0 - E_0)^2}{4kR\mathcal{K}}\right]^{1/3} \tag{16}$$

which corresponds to $\kappa(B_{opt}) = 2^{-3/2}$, $K = 2$ and

$$I(B_{opt}) = \frac{U_0 - E_0}{2R} \;\; ; \;\; \eta(B_{opt}) = \left[\frac{1}{2k} - \frac{E_0}{2kU_0}\right]. \tag{17}$$

One can also check if an optimum electric field exists, maximising the thruster efficiency. Solving $\partial_{U_0}\eta$, an explicit expression for $U_0^{\eta_{max}}$ is obtained. Assuming $\tilde{\nu} = 0$, the expression reduces to

$$U_0^{\eta_{max}} = 2E_0 + k\sqrt{E_0(BH)^3/(\mathcal{K}R)}, \tag{18}$$

leading to a maximum efficiency of

$$\eta_{max} = \max_{U_0} \eta = \frac{1}{k + 2\sqrt{E_0\mathcal{K}R/(BH)^3}}. \tag{19}$$

For this particular voltage, the current and velocity are

$$I^{\eta_{max}} = E_0/R \;\; ; \quad , u_d^{\eta_{max}} = \sqrt{E_0 BH/(\mathcal{K}R)}, \tag{20}$$

giving the load factor

$$K = 1 + \sqrt{E_0\mathcal{K}R/(k^2(BH)^3)} \tag{21}$$

Now, using B_{opt}, the couple (B, U_0) which simultaneously maximise the velocity and the efficiency is

$$U_0^{max} = 3E_0 \quad ; \quad B^{max} = (E_0\mathcal{K}R)^{1/3}/(k^{2/3}H), \tag{22}$$

which corresponds to $\kappa(B_{opt}) = 2^{-3/2}$, $K = 2$ and

$$I^{\eta_{max}} = \frac{E_0}{R} \;\; ; \;\; u_d^{\eta_{max}} = \left(\frac{E_0^2}{k\mathcal{K}R}\right)^{1/3} \;\; ; \;\; \eta = \frac{1}{3k} \tag{23}$$
