# Supplementary material for: Experimental and theoretical study of magnetohydrodynamic ship models
Source: PLoS One. 2017 Jun 30;12(6):e0178599. doi: 10.1371/journal.pone.0178599 (PMC5493298; doi:10.1371/journal.pone.0178599)
Supplement: S4 Appendix — (ZIP) [file pone.0178599.s004.zip › S4_Appendix.pdf]

Mean field of a cylindrical magnet

Focusing on the variation of magnetic field B with the width of the thruster W, fringing effects can be neglected, and a simple model is chosen to study how B evolves with W: the magnetic field is assumed to be the one on the main axis Oz of a cylindrical magnet, i.e. of a solenoid, of radius a and length L_z

$$\frac{B_z}{B_r} = \frac{z/2 + L_z/4}{\sqrt{a^2 + (z + L_z/2)^2}} - \frac{z/2 - L_z/4}{\sqrt{a^2 + (z - L_z/2)^2}}, \tag{1}$$

where B_r is the limit of (1) when $L_z \to \infty$ (i.e. $\lim_{L_z \to \infty} B_z = B_r$). In this limit $L_z \to \infty$, the field $B = B_r$ given by Eq (1) corresponds to the uniform field within the magnet/solenoid (for such an infinite magnet/solenoid, the field outside is simply 0), which shows that B_r is indeed the residual flux density (or induction) for a magnet (magnetic circuit closed at infinity, see section 3).

Now, the magnet mean field $B =< B_z >$, which is B_z averaged on a distance W, is

$$\begin{aligned} B &= \frac{1}{W} \int_{L_z/2}^{W + L_z/2} B_z \,\mathrm{d}z \\ &= \frac{B_r}{2W} [a + \tilde{a} - \sqrt{a^2 + L_z^2} - \sqrt{a^2 + W^2}], \end{aligned} \tag{2}$$

using $\tilde{a} = \sqrt{a^2 + (W + L_z)^2)}$ and B_z given by Eq (1). Eq (2) shows that B is constant for small W, but decreases as $1/W$ for large W. The critical value W_c below which B is nearly constant can be estimated by solving $B_{(W \to 0)} = B_{(W \to \infty)}$. Noting $x = a/L_z$, we obtain $W_c/a = -x - 1/x + \sqrt{1 + x^2} + \sqrt{1 + 1/x^2} = f(x)$, i.e. $W_c \approx a$ since $f(x)$ is nearly constant, around 1 (which is confirmed by the limits for $x \to 0$ and $x \to \infty$, both equal to 1).

In the usual limit $L_z \ll a$, a compact approximation of Eq (2) is

$$B = \frac{B_r}{2} \frac{L_z}{a + W}, \tag{3}$$

which agrees with the two asymptotic expressions of Eq (2) for small and large W, and allows to recover $W_c \approx a$. Equations (2), or its approximation (3), are half the mean field created between two aligned attracting cylindrical magnets.
